# Potentiometric Sensor Arrays Based on Hybrid PFSA/CNTs Membranes for the Analysis of UV-Degraded Drugs

**DOI:** 10.3390/polym15122682

**Published:** 2023-06-14

**Authors:** Anna Parshina, Anastasia Yelnikova, Ekaterina Safronova, Tatyana Kolganova, Olga Bobreshova, Andrey Yaroslavtsev

**Affiliations:** 1 Department of Analytical Chemistry, Voronezh State University, 394018 Voronezh, Russia; anastasia_elnikova@outlook.com (A.Y.); tanyadenisova@list.ru (T.K.); bobreshova@chem.vsu.ru (O.B.); 2 Kurnakov Institute of General and Inorganic Chemistry RAS, 119991 Moscow, Russia; safronova@igic.ras.ru (E.S.); yaroslav@igic.ras.ru (A.Y.)

**Keywords:** Donnan potential, potentiometric multisensory system, PFSA, functionalized CNTs, hybrid material, ultrasonic treatment, ion transport, sulfacetamide, sulfanilamide, degraded drug

## Abstract

The degradation of drugs is a substantial problem since it affects the safety and effectiveness of pharmaceutical products, as well as their influence on the environment. A novel system of three potentiometric cross-sensitive sensors (using the Donnan potential (DP) as an analytical signal) and a reference electrode was developed for the analysis of UV-degraded sulfacetamide drugs. The membranes for DP-sensors were prepared by a casting procedure from a dispersion of perfluorosulfonic acid (PFSA) polymer, containing carbon nanotubes (CNTs), whose surface was preliminarily modified with carboxyl, sulfonic acid, or (3-aminopropyl)trimethoxysilanol groups. A correlation between the sorption and transport properties of the hybrid membranes and cross-sensitivity of the DP-sensor to sulfacetamide, its degradation product, and inorganic ions was revealed. The analysis of the UV-degraded sulfacetamide drugs using the multisensory system based on hybrid membranes with optimized properties did not require a pre-separation of the components. The limits of detection of sulfacetamide, sulfanilamide, and sodium were 1.8 × 10^−7^, 5.8 × 10^−7^, and 1.8 × 10^−7^ M. The relative errors of the determination of the components of the UV-degraded sulfacetamide drugs were 2–3% (at 6–8% relative standard deviation). PFSA/CNT hybrid materials provided the stable work of the sensors for at least one year.

## 1. Introduction

The degradation of the active ingredients of pharmaceutical drugs and the presence of related organic impurities in their content are serious problems. This is due to the fact that some side components may greatly affect the safety and effectiveness of drugs. High-performance (HP) liquid chromatography (LC) with UV detection [1,2], reversed-phase HPLC with photodiode array [3,4,5], UV [6,7] or fluorescence [7] detectors, HP thin-layer chromatography with densitometry [1,2,4], capillary zone electrophoresis [8,9,10], and spectrophotometry combined with chemometrics [11,12,13,14,15,16] can be used to control the degradation and purity of drugs. At the same time, there are two main approaches to the analysis. In the first case, active ingredients are determined in the presence of side components so that the content of the latter does not influence an analytical signal [1,2,3,8,11,12]. The second approach is the simultaneous determination of drug components, including related organic impurities and degradation products [4,5,6,7,9,10,13,14,15,16].

With regard to rapidity, high precision in a wide range of concentrations, low reagent consumption, relatively low cost, and convenience, electrochemical sensors are attractive for the analysis of pharmaceutical drugs.

Voltammetric sensors both for the determination of active ingredients in the presence of side components and for their simultaneous determination are known. For example, voltammetric sensors based on glass carbon electrodes, modified by a nanocomposite of nitrogen-doped hollow carbon nanospheres wrapped with MoS_2_ nanosheets [17] or by a chitosan-covered hybrid composite based on reduced graphene, palladium, and gold nanoparticles [18], were developed for the simultaneous determination of acetaminophen and its primary hydrolytic degradation product (4-aminophenol). Paste electrodes are widely used for the development of voltammetric sensors. A paste electrode made of glass carbon nanospheres containing a CeO_2_–ZnO–chitosan composite provided the simultaneous determination of acetaminophen and 4-aminophenol as well [19]. Carbon paste electrodes modified with C18 silica were described for the simultaneous determination of lidocaine and its metabolite (2,6-dimethylaniline) [20]. Paste electrodes based on carbon nanotubes (CNTs) were developed for the determination of butenafine [21] and naproxen [22] in the presence of their degradation products. The selective determination of lurasidone in the presence of its hydrolytic and oxidative degradation products was achieved using a screen-printed electrode made of graphite ink containing FeO nanoparticles and reduced graphene oxide [23]. The absence of the interfering influence of potential hydrolytic degradants of new carbapenems (meropenem and ertapenem) was revealed using a pencil graphite electrode modified with poly (bromocresol green) [24].

The potentiometric selective determination of active ingredients in pharmaceutical drugs is possible mainly due to the introduction of ion pairs of analytes into a poly (vinyl chloride) (PVC) membrane. Therefore, a sensor with an inner reference solution based on tetrakis (4-chlorophenyl) borate used as an ionophore was developed for the selective determination of eszopiclone [25]. The same cation exchanger along with 2-hydroxy propyl-β-cyclodextrin were introduced into the PVC membrane of solid contact potentiometric sensors for the determination cyclopentolate hydrochloride and phenylephrine hydrochloride in the presence of their alkali and oxidative degradation products, respectively [26]. Solid contact sensors, prepared by the traditional method and by screen-printing, showed high selectivity to bromazepam owing to the use of the PVC membrane containing ion pairs of the analyte with tetraphenylborate [27].

The simultaneous determination of the key and side components of drugs using different methods is complicated by the presence of the same structural moieties of the analytes, which are relevant to the formation of the analytical signal. This makes it difficult to discriminate between the responses for each component of the analysis object. The use of multidimensional mathematical methods for data processing may be the solution to the problem. This approach is used in spectrophotometry very successfully [13,14,15,16]. In the case of electrochemical methods, chemometric techniques are generally combined with arrays of cross-sensitive sensors. This is called a multisensory approach. It allows the use of nonspecific sensor materials, which widens the ranges of materials used for the sensor development and analytes for the determination, as well as the range of the performing analytical goals [28,29].

It seems prospective to adopt materials from other applications for the sensor development. Therefore, the interest in perfluorosulfonic acid (PFSA) membranes and hybrid materials based on them has not been diminishing for a long time. The PFSA membranes are made of a hydrophobic perfluorinated polymer matrix and a system of hydrophilic pores and channels. The main functional properties of the PFSA membranes (high selectivity to cations and conductivity) are defined by the hydrophilic phase [30,31,32]. The change in the conditions for the film formation [33,34,35,36] and their subsequent treatment [37,38], as well as the introduction of dopants of different natures [39,40,41,42] allows us to vary the sorption and transport properties of the PFSA membranes. The chemical and mechanical stability of the materials based on the PFSA polymers is no less important. Such materials are widely used in hydrogen–air fuel cells [43], lithium metal batteries [44], vanadium redox flow batteries [45], and water treatment [46]. The combination of the stated properties is also the reason for the use of the PFSA membranes in electrochemical sensors. They are used for the dispersion [47,48,49] and stabilization [50,51,52] of active components of the sensors, as well as a barrier to interfering components (for instance, for protection against organic pollutants or oxidant anions) and for the analyte concentration [53,54,55].

The PFSA membranes modified with CNTs are hybrid materials with a mixed electron-ionic conductivity and high sorption ability [56,57]. The use of such materials in voltammetric sensors provides a simultaneous increase in the sensitivity and selectivity, as well as a reduction in the exposure to fouling [58,59]. The modification of the CNTs’ surface by hydrophilic groups allows us to decrease their aggregation in the PFSA polymer dispersion and affects their distribution between the hydrophilic and hydrophobic membrane phases. It provides an opportunity to change the permeability and sorption capacity of the membranes for the analytes of a different nature. In our previous work [60], the nature varying of functional groups on the surface of CNTs, which were introduced into the PFSA membranes, provided the development of an array of potentiometric cross-sensitive DP-sensors (using the Donnan potential (DP) as an analytical signal) for the analysis of the combination drug of sulfamethoxazole and trimethoprim. It seems very interesting to study such materials for the simultaneous determination of the 4-aminobenzenesulfonic acid derivatives (including sulfacetamide (SAA)) with the primary member of the class (sulfanilamide (SA)), since it is their main UV-degradation product.

This work was devoted to the development of an array of cross-sensitive DP-sensors based on hybrid materials consisting of the PFSA membranes, used as the main ion-conducting phase, and functionalized CNTs, used as a dopant, for the simultaneous determination of SAA and SA in UV-degraded drugs.

## 2. Materials and Methods

### 2.1. Materials and Reagents

A 10 wt% dimethylformamide (DMF) dispersion of the PFSA polymer with equivalent weight of 1100 in the Li^+^ form (MF-4SC, Plastpolymer, Saint-Petersburg, Russia) and multiwall CNTs (Taunit S12, NanoTechCenter, Tambov, Russia) were used. Nitric acid (special purity, >70%), acetone (reagent grade, >99.75%), hydrochloric acid (special purity, 35–38%), and potassium chloride (reagent grade) were bought in Chimmed (Moscow, Russia). *D*-glucose (Ph Eur, hydrated form, Merck, Darmstadt, Germany), *p*-toluenesulfonic acid (97.5%, Acros Organics, Geel, Belgium), ethanol (95%, Ferein, Minsk, Belarus), and 3-aminopropyl)trimethoxysilane (97%, Alfa Aesar, Ward Hill, MA, USA) were used. N-[(4-aminophenyl)sulfonyl]acetamide (>99%) and 4-aminobenzenesulfonamide (>99%) were supported from Sigma-Aldrich (Saint-Louis, MO, USA). Sulfacyl sodium-SOLOpharm eye drops (Grotex, Saint-Petersburg, Russia) were analyzed. The deionized water with resistance 18.2 MΩ and pH 5.41 ± 0.05 was obtained using a Simplicity^®^ Water Purification System (MerckMillipore, Darmstadt, Germany).

### 2.2. Functionalization of CNTs

CNT functionalization was performed according to the methods described elsewhere [60]. Conditions for the surface modification of CNTs are listed in Table 1. Commercial CNTs were treated with an oxidant to purify them from the catalyst remains used in their synthesis. This led to the breaking of the C-C bonds on the inner surface of CNTs and the formation of carboxyl and hydroxyl groups. The purified CNTs were treated with a mixture of *p*-toluenesulfonic acid and *D*-glucose under hydrothermal conditions. As a result, a sulfonated polymer, which was bound to the CNT surface by stacking interactions, was synthesized [61]. The carboxyl groups on the CNT surface were the centers for the amination. To increase their concentration, the purified CNTs were additionally treated with the oxidant solution upon obtaining CNTs-NH_3_^+^. As a result of the interactions of carboxyl groups and (3-aminopropyl)trimethoxysilane, -O-Si(OCH_3_)_2_-(CH_2_)_3_-NH_3_^+^ groups, covalently bound to CNTs, were formed.

The IEC and FTIR spectra characteristics, proving the effectiveness of the CNT modification, as well as their structural characteristics (the values of the outer diameter, established using SEM and declared by the manufacturer, were the same) are presented in Table 2. A comprehensive discussion of the data was presented in our previous works [56,57,60].

The designations of CNTs-COO^−^, CNTs-NH_3_^+^, and CNTs-SO_3_^−^ (or CNTs-X in general) were introduced for the functionalized CNTs.

### 2.3. Membrane Synthesis

Hybrid membranes were fabricated by casting procedure from a DMF dispersion of CNTs-X and PFSA polymer [60]. CNTs-X for the dispersions were prepared preliminarily according to the aforementioned methods. The concentration of CNTs-X in the dispersion was 0.5, 1.0, or 1.5 wt% of the polymer weight. To prepare the dispersion, ultrasonic (US) treatment with an RK-100 cleaner sonication bath (Bandelin Electronic, Berlin, Germany) was used. US treatment led to a decrease in viscosity of the dispersions (Table 3) due to deagglomeration and reduction in the average molecular weight of the polymer [62]. A special cell was used for the membrane casting [60]. The PFSA polymer dispersion without US treatment and the US-treated dispersion of CNTs-X and the PFSA polymer were simultaneously cast on a glass surface toward each other. The fabricated film included unmodified and bulk modified parts (gradient modification) with a narrow intermediate part between them. The latter was formed as a result of mixing of the dispersions with and without the dopant. The removal of the solvent was performed using a vacuum-drying oven (Jeio Tech, Daejion, Korea). The films were hot-pressed to improve their endurance. The obtained membranes were conditioned and converted into the K^+^ form. Conditions for every step of the membrane synthesis and preparation for work are listed in Table 3.

Additionally, uniformly modified PFSA/CNTs-X membranes were synthesized for the estimation of the sorption properties and diffusion permeability. Membranes prepared from the PFSA dispersion without US treatment and the PFSA dispersion with US treatment were the reference samples (Table 3).

The designations of the PFSA/CNTs-COO^−^, PFSA/CNTs-NH_3_^+^, and PFSA/CNTs-SO_3_^−^ (or PFSA/CNTs-X in general) were introduced for the hybrid membranes.

### 2.4. Apparatus and Experiment

The ion-exchange capacity (the IEC values are given per 1 g of a dry membrane or the CNTs-X powder) of the materials was determined by titration. The end of the titration was detected using an Ekonix-Expert 001 pH-meter (Ekonix-Expert, Moscow, Russia). Preliminarily, the membranes, conditioned at 150 °C for 30 min, were weighed. The weighed samples of 0.1–0.2 g of the PFSA membranes in the dry state or the powder of CNTs-X were used for the experiments. The PFSA membranes were placed into 50 mL of a 0.1 M NaCl solution, while the powder of CNTs-COO^−^ or CNTs-SO_3_^−^ in 10 mL of a 0.5 M NaCl solution, and CNTs-NH_3_^+^ in 10 mL of a 0.1 M HCl solution. The titration was conducted after 4 h using a 0.05 M NaOH solution.

The change in the membrane weight upon heating from 20 °C to 150 °C was established for the estimation of the water uptake. A Netzsch-TG 209 F1 thermal balance (Netzsch, Selb, Germany) was used. The amount of water molecules per 1 sulfonic acid group (*n*(H_2_O/–SO_3_^−^)) was calculated using the membrane IEC and water uptake.

The diffusion permeability was studied at ~25 °C for a membrane dividing 0.02 M KCl and 0.5 M KCl solutions. The composition of the solutions was controlled by performing conductometric measurements using an Ekonix-Expert 002 conductometer.

A Vertex 70 FTIR spectrometer (Bruker, Mannheim, Germany) was used for the analysis of solid substances of the analytes, their solutions, and solutions of the drugs. Measurements for solid substances were performed relative to air, while, for drug solutions, the measurements were performed relative to deionized water. Additionally, FTIR spectrum of the solution of the UV-degraded drug was obtained relative to the untreated drug solution. FTIR spectra of deionized water and aqueous solutions were registrated in attenuated total reflection mode.

A Shimadzu UV-1800 spectrometer (Shimadzu, Kyoto, Japan) was used for the spectrophotometric analysis of the drugs.

A DP-sensor consisted of a reference electrode, a reference solution (1 M KCl), and an ion-selective membrane in the K^+^ form, which connected the reference and test solutions as a bridge [63].

The depth of membrane immersion into the solutions was no greater than 0.3 cm while its length was 6 cm. A significant increase in the distance between membrane interfaces with the solutions eliminated transmembrane transfer and minimized the diffusion potential. A statistically constant value of the circuit voltage was established in several seconds. The time of the electrolyte diffusion through the membrane (taking into account the membrane permeability of P ≈ 10^−7^ cm^2^/s) exceeded the time of quasi-equilibration significantly. Therefore, the diffusion potential in the membrane phase consisted of two diffusion potentials in the surface layers of the membrane parts which were in contact with the solutions. The diffusion potential in the membrane surface layer, which contacted the concentrated reference solution, was eliminated owing to the concentration closeness of the outer and inner solutions. The DP at this interface was low because of the same reason. The concentration equilibration of the ions at the membrane interface with the diluted test solution was limited by the Donnan exclusion. Thus, the main contribution in the circuit voltage was the DP at the membrane interface with the test solution.

To provide the aforementioned conditions for the measurement of the DP-sensor analytical signal, the membranes were gradient-modified. The end of the modified membrane part was in contact with the test solution while the unmodified one was in contact with the reference solution. As a result of that, the DP at the membrane interface with the reference solution was independent from the dopant concentration and nature. The membrane modified and unmodified parts shared a thin intermediate part (no thicker than 0.5 cm). It did not affect the DP-sensor analytical signal, since the composition of the membrane during the measurement changed only in the surface layer at the interfaces with the solutions.

For the simultaneous estimation of the DP at the interfaces of several membranes with the test solution, the method, described elsewhere [63], was used. The cell included one section for the test solution connected with membranes to several sections for the reference solutions. The cell construction provided the measurement simultaneously for up to 8 membranes. The voltage values between a reference electrode, contacting the test solution, and each of reference electrodes, contacting the reference solution, were measured using an analog-to-digital transmitter multichannel potentiometer. Additionally, the pH of the test solutions was measured. The silver chloride electrodes (ESr-10103) and the glass electrode (ES-10301/4) from Econix-Expert were used.

### 2.5. Model Solutions and Pharmaceutical Solutions

The analytes were SAA and its primary UV-degradation product SA (Equation (1)). Some characteristics of SA and SAA are listed in Table 4.

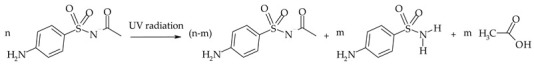
(1)

Precisely weighed amounts of pure substances were used to prepare calibration solutions. The model solutions for potentiometry contained SAA^−^ anions, SA molecules, Na^+^ cations, and water dissociation products (pH 4.68–10.56). The concentrations of SA, SAA, and NaOH were in the range of 1.0 × 10^−5^–1.0 × 10^−3^ M. For the spectrophotometric technique, the model solutions contained SAA molecules with concentrations from 1.0 × 10^−5^ to 4.0 × 10^−5^ M and SA molecules with concentrations from 0.2 × 10^−5^ to 0.8 × 10^−5^ M. Moreover, an acetate buffer solution (pH = 4.0) was added.

The drug was subjected to forced degradation. The initial composition of the Sulfacyl sodium-SOLOpharm eye drops included SAANa·H_2_O (the SAANa concentration is 200 mg/mL), Na_2_S_2_O_3_·5H_2_O (1.0 mg/mL), HCl (for the pH correction), and purified water. The drug was diluted 500 times with deionized water and treated with UV radiation for 10 min. To perform potentiometric and spectrophotometric measurements, additional 2-fold and 50-fold dilutions were required, respectively. To perform the spectrophotometric analysis of the untreated drug, it was diluted 25,000 times.

The data on the conditions of SAA degradation with the formation of SA were found in literature [66]. Additionally, the analysis of the drug before and after forced degradation was performed by FT-IR spectroscopy.

**Figure 1:**
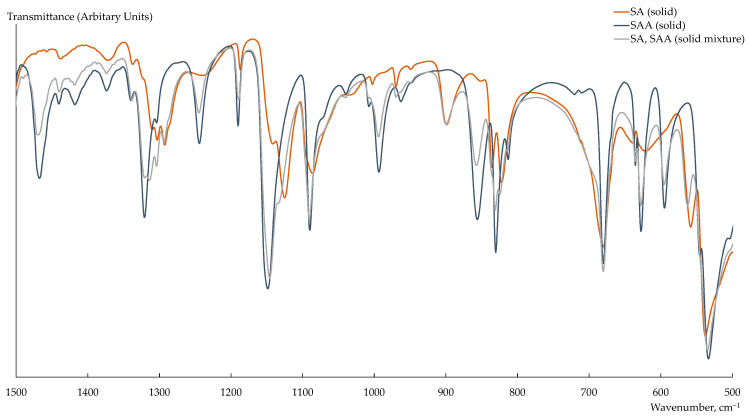
FTIR spectra of solid substances of SAA, SA, and their mixture.

**Figure 2:**
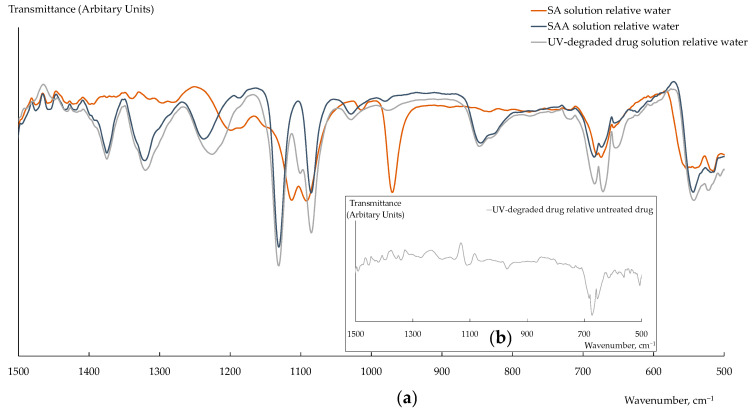
FTIR spectra of solutions of SA (0.8 M), SAA (0.8 M), and the UV-degraded drug (dilution 1/10) relative deionized water (**a**) and the UV-degraded drug relative untreated drug (dilution 1/10) (**b**).

The FTIR spectrum of the solid mixture of SAA and SA differed from that of SAA in the form and intensity of the peaks in the following regions (Figure 1): ~1470 cm^−1^ (CH_3_ bending vibrations), ~1300 cm^−1^ (SO_2_ asymmetric stretching vibrations), ~1130 cm^−1^ (SO_2_ symmetric stretching vibrations), ~1250 cm^−1^ (N–H bending vibrations and C–N stretching vibrations), and ~620 cm^−1^ (O=C–N bending vibrations). Moreover, a new band at 900 cm^−1^ (S–N stretching vibration), which is characteristic of SA and is not observed in the SAA spectrum due to the presence of an acetate substitute of nitrogen atom, appeared (Figure 1). The FTIR spectra of the aqueous SAANa and SA solutions, established relative to deionized water, had slightly pronounced differences and low intensity of the peaks (Figure 2). With a small reliability, it may be said that, in the FTIR spectrum of the UV-degraded drug solution comparing to that of the SAANa solution, the form of the peak in the 1100 cm^−1^ region (SO_2_ symmetric stretching vibrations) changed because of the decrease in the SAA concentration and the lack of the corresponding peaks for SA (Figure 2a). Moreover, in the FTIR spectrum of the UV-degraded drug solution, established relative to deionized water, the band with a low intensity at 974 cm^−1^ (S–N stretching vibrations) and characteristic of the SA solution appeared. The appearance of the band at 970 cm^−1^ was also observed in the FTIR spectrum of the UV-degraded drug solution, established relative to the untreated drug solution (Figure 2b). The low intensity of the peak in this region was due to the low concentration of SA formed upon degradation.

### 2.6. Data-Processing Procedures

The DP-sensor calibration characteristics were calculated by the least-square method according to the algorithm described previously [67]. The dependence of the DP-sensor analytical signal was described by a multidimensional linear regression equation with a statistically insignificant scatter between theoretically predicted and experimental values in the studied concentration range (Equation (2)). The response reproducibility was estimated as a mean value of the response variance for the matrix of the calibration equations:

(2) $${\Delta \varphi }_{D}=b_{0}+b_{1}\times pNa+b_{2}\times pH+b_{3}\times pSA+b_{4}\times pSAA,$$

where ∆*ϕ_D_* (mV) is the DP-sensor analytical signal; pNa, pSA, and pSAA are negative decimal logarithms of the Na^+^, SA, and SAA^−^ concentration, respectively; and *b_i_* (mV/p*c*) is the sensitivity coefficient to the Na^+^, SA, and SAA^−^, respectively.

A sensory array for the analysis of the degraded drug included three DP-sensors with a high sensitivity to the analytes and minimal correlation between the analytical signals. Moreover, a glass electrode was added into the multisensory system to provide an opportunity to perform the analysis without pH correction. A system of three calibration equations was used for the simultaneous determination of Na^+^, SA, and SAA^−^. The “3σ” rule was used for the estimation of limits of detection (LODs) of the analytes.

**Figure 3:**
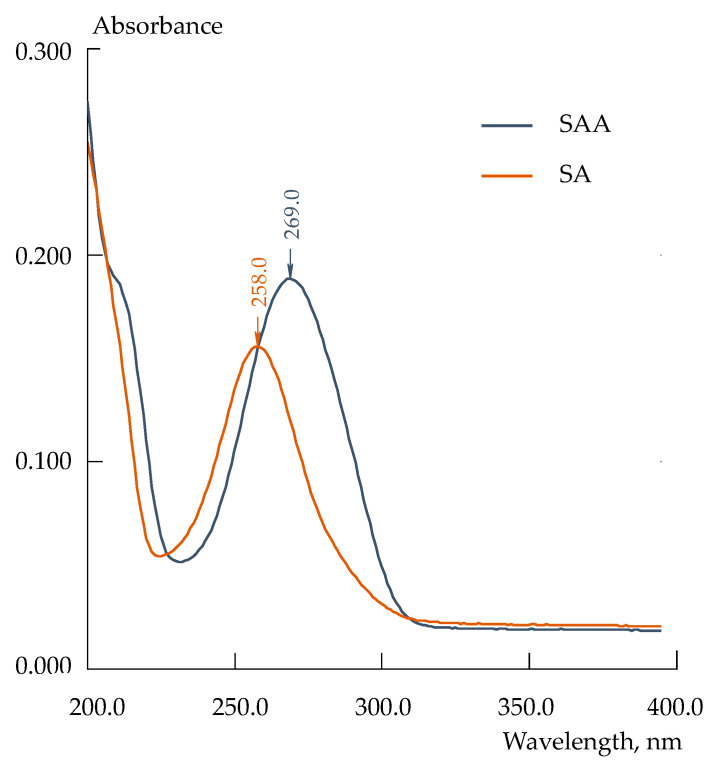
UV spectra of absorbance of 1.0 × 10^−5^ M SAA and 0.8 × 10^−5^ M SA solutions at pH = 4.0.

To perform the spectrophotometric analysis of the initial and UV-degraded drug, calibrations of Equations (3) and (4) were obtained, respectively. A multidimensional regression analysis was used because a satisfactory resolution of the characteristic peaks of SAA and SA, which are structural analogs, in their UV spectra was not achieved (Figure 3).

(3) $$A_{269}=16.8\times 10^{3}\cdot c_{SAA}$$

(4) $$\left\{\begin{array}{c} A_{258}=12.5\times 10^{3}\times c_{SAA}+16.6\times 10^{3}\cdot c_{SA},\\ A_{269}=15.9\times 10^{3}\cdot c_{SAA}+14.8\times 10^{3}\cdot c_{SA}, \end{array}\right.$$

where A*_i_* is the absorbance at the *i*-th wavelength. The wavelengths 258 and 269 nm corresponded to the absorbance maxima of individual solutions of SA and SAA, respectively.

## 3. Results and Discussion

### 3.1. Cross-Sensitivity of the DP-Sensors

**Figure 4:**
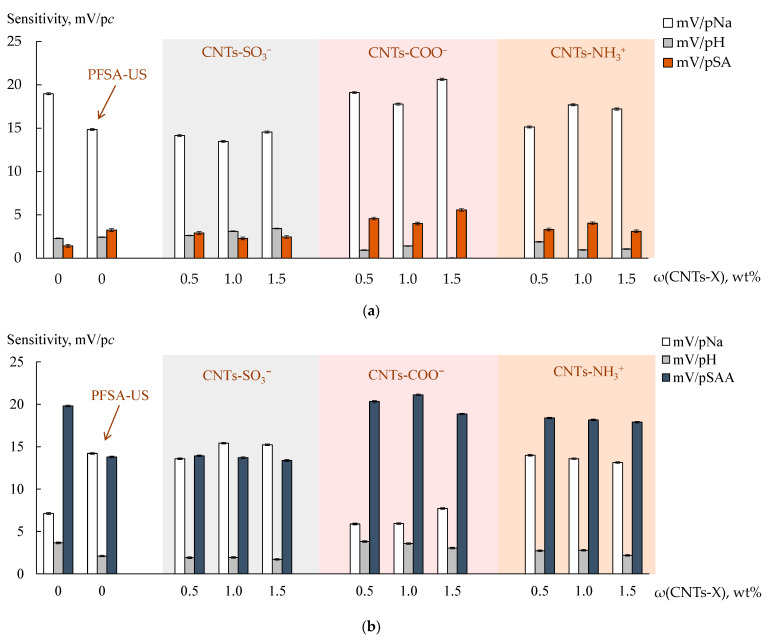
DP-sensor sensitivity to ions in the solutions of SA + NaOH (**a**) and SAA + NaOH (**b**) for the PFSA, PFSA-US, and PFSA/CNTs-X membranes.

The sensitivity of DP-sensors based on the PFSA and PFSA-US membranes in the K^+^ form to SA molecules was 1.43 ± 0.11 and 3.25 ± 0.14 mV/p*c*, while, to SAA^−^ anions, it was 19.82 ± 0.11 and 13.79 ± 0.08 mV/p*c*, respectively (Figure 4). Both analytes entered the membrane by non-exchange sorption. The sensitivity to SAA^−^ anions was significantly higher than to SA molecules despite the effect of the Donnan exclusion. This was due to the higher hydrophilicity of SAA^−^ anions (Table 4), as well as their facilitated mechanism of the transfer into the membrane. The latter was owing to the presence in the solutions of the other mineral ion (Na^+^) instead of the initial ionic form of the membrane (K^+^) [63]. SAA^−^ anions entered the membrane due to the formation of hydrogen bonds between the hydration shells of the charged sulfonamide moieties and the hydration shells of the counter-ions. The formation of hydrogen bonds between aniline amino groups of the analytes and sulfonic acid groups of the membrane was also possible.

**Figure 5:**
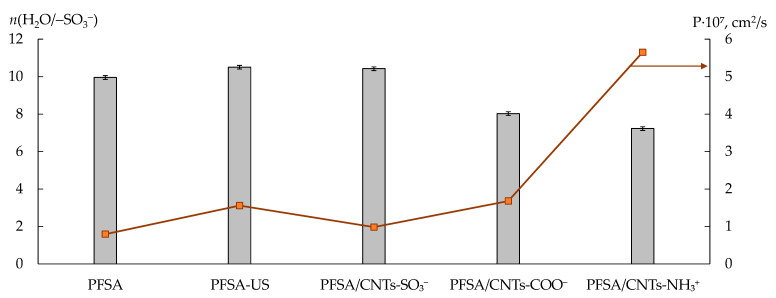
The amount of water molecules per 1 sulfonic acid group (*n*(H_2_O/–SO_3_^−^)) in contact with water and diffusion permeability (the relative error is below 1.0%) of the membranes in the K^+^ form (the CNTs-X concentration was 1.0 wt%).

The dopant in the PFSA/CNTs-X membranes was mainly located in the hydrophobic matrix and partially located in the hydrophilic phase owing to the functionalization of the surface [60]. The presence of the dopant in the pores was proven by an increase in the IEC of the PFSA/CNTs-SO_3_^−^ membranes of up to 1.03 mmol/g and a decrease in that of the PFSA/CNTs-NH_3_^+^ membranes of down to 0.94 mmol/g (the values are for the dopant concentration of 1.5 wt%) in comparison with the IEC of the initial membrane (1.00 mmol/g). The IEC of the PFSA/CNTs-COO^−^ and PFSA-US membranes was the same (0.97–0.98 mmol/g) and slightly below the initial one. The latter was due to the loss of some –SO_3_^−^ groups as a result of the breaking of the polymer chains upon US treatment of the polymer dispersion [60]. The dopant parts located in the pores could be the centers for the electrostatic and stacking interactions with the analytes. The affinity of the dopant functional groups to the analytes increased in the membrane series of PFSA/CNTs-SO_3_^−^ < PFSA/CNTs-COO^−^ < PFSA/CNTs-NH_3_^+^. An increase in the membrane permeability to non-exchange sorbed particles (the diffusion permeability) was observed in the same series (Figure 5). The rate of anion transfer through the PFSA/CNTs-SO_3_^−^ membrane (when the dopant concentration was no greater than 1.0 wt%) was lower than through the PFSA-US membrane due to the higher concentration of the proton-acceptor groups which prevented the anion transfer within the pores. The diffusion permeability of the PFSA/CNTs-COO^−^ membranes was comparable to that of the PFSA-US membrane. The introduction of the anion-exchange groups and a partial binding of the –SO_3_^−^ groups led to a sufficient increase in the diffusion permeability of the PFSA/CNTs-NH_3_^+^ membranes. Moreover, in the work [60], it was proposed that CNTs-NH_3_^+^ were incorporated into the coils of the polymer macromolecules in the dispersion because of the amphiphilic nature of both of them and the presence of the opposite-charged groups. As a result, a well-developed system of pores and channels of the membrane was formed, and the CNTs-NH_3_^+^ concentration in the hydrophilic phase was increased. This was also favored by a higher level of the surface functionalization of CNTs-NH_3_^+^ compared to CNTs-COO^−^ and CNTs-SO_3_^−^. At the same time, the sensitivity of DP-sensors to SA molecules and SAA^−^ anions increased in the membrane series of PFSA/CNTs-SO_3_^−^ < PFSA/CNTs-NH_3_^+^ < PFSA/CNTs-COO^−^ (Figure 4). It is due to the fact that the CNT surface, containing a small amount of covalently bound carboxyl groups, provides higher availability to the analytes, whereas –NH_3_^+^ groups on the surface of CNTs were included in the bulk moieties, which screened the CNT surface, and were partially bound to the membrane –SO_3_^−^ groups.

The influence of the membrane modification on the DP-sensor sensitivity to Na^+^ cations depended on the nature of the organic analyte, presented in the solutions. The sensitivity of DP-sensors in the solutions, containing SA molecules, to Na^+^ cations increased in the series of PFSA/CNTs-SO_3_^−^ < PFSA-US < PFSA/CNTs-NH_3_^+^ < PFSA < PFSA/CNTs-COO^−^ (Figure 4). It correlated with the change in the amount of water molecules per one –SO_3_^−^ group in the membranes in the series of PFSA/CNTs-SO_3_^−^ > PFSA-US > PFSA > PFSA/CNTs-COO^−^ > PFSA/CNTs-NH_3_^+^, except for the position of the PFSA/CNTs-NH_3_^+^ membrane (Figure 5). Some decrease in the IEC and a significant reduction in the water uptake of the PFSA/CNTs-COO^−^ and PFSA/CNTs-NH_3_^+^ membranes led to an increase in the molar concentration of the counter-ions in the membranes and an increase in the DP-sensor sensitivity to them. However, the increase in the DP-sensor sensitivity to Na^+^ cations was weakly pronounced for the PFSA/CNTs-NH_3_^+^ membrane since its diffusion permeability increased and should decrease in the counter-ion transport numbers in the membrane (Figure 5). In the solutions, containing SAA^−^ anions, the DP-sensor sensitivity to Na^+^ cations decreased with the increasing sensitivity of DP-sensors to the organic analyte. This was an additional proof of the sufficiently high affinity of the membranes to SAA^−^ anions, the presence of which, in the membrane pores, might decrease the availability of the functional groups for the ion exchange.

### 3.2. Characteristics of the Multisensory System

**Figure 6:**
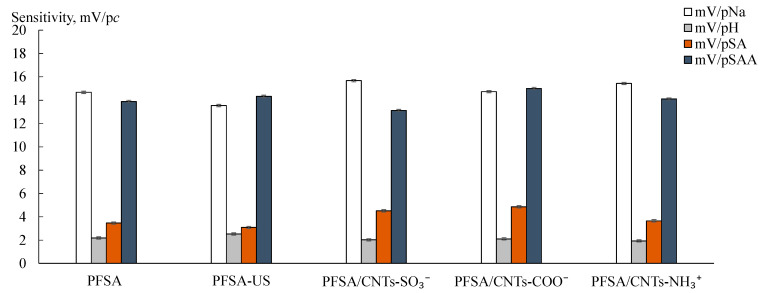
DP-sensor sensitivity to ions in the solutions of SA + SAA + NaOH for the PFSA, PFSA-US, and PFSA/1.0 wt% CNTs-X membranes.

The DP-sensor sensitivity for all membranes to SA molecules and SAA^−^ anions in the solutions, containing both analytes, was lower (Figure 6). For these solutions, the dependencies discussed above were the same. Nevertheless, they were less pronounced than when studying the solutions containing just one of the organic analytes and the alkali (Figure 4). It seemed that the reason was the steric factor limiting the sorption of the bulk organic analytes.

DP-sensors based on the PFSA-US, PFSA/1.0 wt% CNTs-COO^−^, and PFSA/1.0 wt% CNTs-SO_3_^−^ membranes were included into a multisensory system for the analysis of aqueous solutions, containing SAA^−^, SA, and Na^+^ as analytes (Table 5). The PFSA/1.0 wt% CNTs-COO^−^ membrane was chosen since it provided the highest sensitivity of the DP-sensor to both organic analytes due to the higher availability of the sorption centers. Two other membranes were selected so that the correlation between the responses of the DP-sensors based on them and the response of the DP-sensor based on the PFSA/1.0 wt% CNTs-COO^−^ membrane was the lowest.

The composition of the membranes and their preparation conditions did not significantly affect the time of the DP-sensor response. Using chronopotentiometry, it was established that the change of the response value after several seconds of the DP-sensor contact with the analyte solution was no greater than the scatter upon repetition of the experiment. The drift of the DP-sensor response after quasi-equilibration was insignificant or varied from 4 to 8 mV/h. The DP-sensor calibration was repeated for several weeks after membrane preparation (the results are presented in Figure 6). The variance of the DP-sensor response (*D*, mV^2^) was 16–19 mV^2^. The scatter (*ε*, mV) between theoretically predicted and experimental values varied from 6 to 7 mV. After a long-term work with the membranes, a recalibration of the multisensory system (the comparison of the initial and re-established calibrations is given in Table 5) was carried out. This allowed us to conclude that degradation and fouling of the membranes after a long period of their use in the DP-sensors were not observed if the storage and operation conditions were proper (Table 6). The stability of the sensory characteristics was due to a number of reasons. Macromolecules of the PFSA polymer were efficiently entangled in the membrane between each other and CNTs-X particles since the membranes were formed by a casting procedure from the dispersions in the aprotic solvent. This prevented the leakage of the dopant from the membrane upon its operation and storage. The treatment of the membranes with KCl solutions effectively purified them from organic analytes, which could be accumulated in the membrane part, contacting the test solution. The sorption of the analytes in the membrane bulk was excluded owing to the original organization of the DP-sensor, minimizing the transmembrane transfer.

The LODs of SAA^−^, SA, and Na^+^ were 1.8 × 10^−7^, 5.8 × 10^−7^, and 1.8 × 10^−7^ M, respectively. The relative errors of the SAA^−^, SA, and Na^+^ determination in the model solutions were 5–10% (at 5–18% RSD), 6–17% (at 4–13% RSD), and 1.7–9% (at 4–20% RSD), respectively (Table 5). The highest RSD values for each analyte were established in solutions, which contained the concentration of the analyte close to the lower limit of the concentration working range.

### 3.3. Analysis of the UV-Degraded Drug

The analysis of the Sulfacyl sodium-SOLOpharm eye drops, subjected to forced degradation, was performed using a multisensory system based on the PFSA-US, PFSA/1.0 wt% CNTs-COO^−^, and PFSA/1.0 wt% CNTs-SO_3_^−^ membranes, as well as by the spectrophotometric method. Using the latter, it was established that the concentration of SAANa in the drug without UV treatment was 200.6 ± 1.2 mg/mL (the error relative to the concentration declared by the manufacturer was 0.5%) while in the UV-degraded drug, it was 184.5 ± 1.7 mg/mL (Table 7). At the same time, the concentration of SA in the UV-degraded drug was comparable with the error of its determination by spectrophotometry. The use of the DP-sensor array provided a statistically significant determination of three analytes in the solution of the UV-degraded drug. The concentrations of SAA^−^, SA, and Na^+^ were (8.1 ± 0.8) × 10^−4^, (6.7 ± 0.8) × 10^−5^, and (8.9 ± 0.8) × 10^−4^ M at a 1000-fold dilution of the drug. Hence, the content of the UV-degraded drug was calculated to be the following: 190 ± 18 mg/mL of SAANa and 11.5 ± 1.4 mg/mL of SA (Table 8).

The relative error of the SAANa determination in the UV-degraded drug using the DP-sensor array relative to the concentration found by spectrophotometry was 3%. Taking into account the fact that the difference in the SAANa concentrations in the initial and UV-treated drug was due to the formation of SA, the error of the SA determination using the DP-sensor array was 2% compared to the spectrophotometry.

### 3.4. Comparison of the Proposed Sensors with the Reported Ones

In our previous works, multisensory systems with cross-sensitive DP-sensors based on the PFSA membranes modified with poly(3,4-ethylenedioxythiophene) (PEDOT) or polyaniline (PANI) were developed for the analysis of drugs of 4-aminobenzenesulfonic acid derivatives [67] (Table 9). One of the stages of the composite membrane preparation was their hydrothermal treatment (HT) [68]. The relative errors and RSD of the determination of the drug components were comparable for the arrays of DP-sensors based on both the PFSA/CNTs-X membranes, and the PFSA/PANI and PFSA/PEDOT membranes. Precursors for the preparation of PANI or PEDOT are cheaper and more available, while varying the conditions of the dopant synthesis provides regulation of their distribution in the membrane and directed changes in the characteristics of devices based on them. At the same time, the PFSA/CNTs-X membranes have some advantages over the composite membranes based on conductive polymers. As already noted, the CNTs-X are located in the matrix of the PFSA membrane. It almost completely eliminates the possibility of dopant leakage from the membrane and improves the mechanical properties of the material. Moreover, the preliminary modification of the CNT surface provides huge opportunities for the fabrication of materials with new properties to different analytes.

To prepare selective potentiometric sensors for the analysis of drugs of 4-aminobenzenesulfonic acid derivatives, we use plasticized PVC membranes or carbon paste containing ion pairs of anion-exchangers with the analytes [69,70,72] or molecularly imprinted polymers (MIPs) [71,73] (Table 9). The selectivity of the sensors based on anion-exchangers to the determining 4-aminobenzenesulfonic acid derivatives in the precense of their derivatives was not estimated [70,72] or was not high (the selectivity coefficient K^Pot^_SAANa,SA_ was 5.63 × 10^−2^ M [69]). Some works showed high selectivity when MIPs are used as ionophores (–logK^Pot^_SMX,SAA_ and –logK^Pot^_SMX,Sulfasalazine_ varied from 3 to 4 depending on the method of their estimation and sensor composition), as well as a wider linear range of concentrations and lower limits of detection [73] (Table 9). At the same time, the authors did not always state how they solve such problems of using MIPs such as the complexity of the bound analyte extraction, the difference in the constants of multiple interaction of the analyte and reaction centers, as well as non-specific binding with the matrix material. It should be mentioned that the selective potentiometric sensors [69,70,71,72,73] have a sufficiently shorter time of stable work than the cross-sensitive DP sensors with the PFSA-based hybrid and composite membranes (Table 9).

## 4. Conclusions

A novel multisensory system, using the Donnan potential (DP) as an analytical signal, was developed for the analysis of UV-degraded sulfacetamide drugs. Membranes for cross-sensitive DP-sensors were prepared by a casting procedure from a dispersion of perfluorosulfonic acid (PFSA) polymer, containing carbon nanotubes (CNTs), which surface was preliminarily modified with –COO^−^, –SO_3_^−^ (in the form of the sulfonated polymer), or –O–Si(OCH_3_)_2_–(CH_2_)_2_–NH_3_^+^ groups. The formation of the membranes from the dispersions of the polymer and the dopant in the aprotonic solvent (DMF) with a preliminary US treatment provided their high stability. A correlation between the sorption and transport properties of hybrid membranes and DP-sensor cross-sensitivity to sulfacetamide, its degradation product, and inorganic ions was revealed. The sensitivity of DP-sensors to SA molecules and SAA^−^ anions increased for the membranes, containing CNTs modified with (3-aminopropyl)trimethoxysilanol (because of the presence of the proton-acceptor groups and the enhanced permeability for anions of the hybrid membranes) and carboxyl (because of the highest availability of the dopant surface for interactions) groups. The analysis of the UV-degraded sulfacetamide drugs using the multisensory system based on hybrid membranes with optimized properties did not require the pre-separation of the components. The limits of detection of sulfacetamide, sulfanilamide, and sodium were 1.8 × 10^−7^, 5.8 × 10^−7^, and 1.8 × 10^−7^ M. The errors of the determination of the components of the UV-degraded sulfacetamide drugs were 2–3% (at 6–8% RSD). The degradation and fouling of the membranes after a long-term use in the DP-sensors were not observed.

## Figures and Tables

**Table 1:** Conditions for the CNT functionalization [60].

Designation	Precursor	Treatment	Drying
CNTs-COO^−^	30 wt% HNO_3_ aqueous solution	CNTs:HNO_3_ (1:8 by weight), 90 °C (1 h), washing with deionized water	in air, 90 °C (24 h)
CNTs-SO_3_^−^	*p*-toluenesulfonic acid, *D*-glucose	CNTs-COO^−^:*p*-toluenesulfonic acid:*D*-glucose (1:1.25:1.25 by weight), hydrothermal treatment at 180 °C (24 h), washing with deionized water and ethanol	in air, 110 °C (24 h)
CNTs-NH_3_^+^	6 M HNO_3_ aqueous solution	CNTs-COO^−^:HNO_3_ (1:8 by weight), 90 °C (1 h), washed with deionized water	in air, 90 °C (24 h)
	5 wt% solution of (3-aminopropyl)trimethoxy- silane in acetone	CNTs-COO^−^:(3-aminopropyl)trimethoxysilane (10:1 by weight), 80 °C (0.5 h), washing with deionized water	in air, 90 °C (24 h)

**Table 2:** Some characteristics of the functionalized CNTs [60].

Designation	CNTs-COO^−^		CNTs-SO_3_^−^		CNTs-NH_3_^+^	
Surface fragments	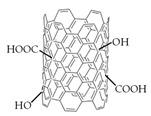		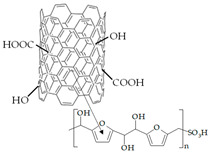		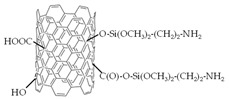	
Functionalization	covalent		non-covalent		covalent	
Outer diameter, nm	20–40		20–40		20–40	
IEC, mmol/g	0.014		0.27		0.64	
FTIR spectra characteristics	1364 cm^−1^	-COOH	986 cm^−1^	-SO_3_H	1056, 899 cm^−1^	-NH_2_
	3700–3500 cm^−1^	-OH	1178 cm^−1^		1196 cm^−1^	Si-O
			3700–3500 cm^−1^	-OH	3700–3500 cm^−1^	-OH

**Table 3:** Conditions for the membrane preparation [60].

Designation	Precursor	Treatment, Viscosity	Film Formation	Drying, Pressing	Conditioning
PFSA	10 wt% DMF dispersion of the PFSA polymer in the Li^+^ form	-, 82.5 ± 0.4 mPa·s	Casting on a glass surface	Drying in vacuum at 60 °C (4 h), 80 °C (12 h), 110 °C (4 h), hot-pressing at 5 MPa, 110 °C (3 min)	5 wt% HCl solution at room temperature (1.5 h), washing with deionized water, 2 M KCl solution (72 h), washing with deionized water
PFSA-US		US, 35 kHz, ≤50 °C (45 min), 64.6 ± 0.6 mPa·s			
PFSA/CNTs-X	DMF dispersion of CNTs-X and PFSA polymer (10 wt%) in the Li^+^ form	US, 35 kHz, ≤50 °C (45 min), 58–62 mPa·s	Casting two dispersions on a glass surface toward each other		
	10 wt% DMF dispersion of the PFSA polymer in the Li^+^ form	-, 82.5 ± 0.4 mPa·s			

**Table 4:** Some characteristics of SA and SAA (*P* is the partition coefficient between octanol and water, and *D*_pH=7_ is the distribution coefficient at pH = 7).

Characteristics	SA	SAA
Dissociation constant	pK_a1_ = 2.40, pK_a2_ = 10.43 [64]	pK_a1_ = 1.78, pK_a2_ = 5.38 [64]
Log *P*	−0.67 [65]	−0.96 [65]
Log *D*_pH=7_	−0.67 [65]	−2.24 [65]
Mole fractions at pH = 7	1.00 SA	0.98 SAA^−^ 0.02 SAA

**Table 5:** The characteristics of the multisensory systems based on the PFSA-US, PFSA/1.0 wt% CNTs-COO^−^, and PFSA/1.0 wt% CNTs-SO_3_^−^ membranes for the determination of SAA^−^, SA and Na^+^ in aqueous solutions.

Characteristic	Membrane Composition		
	PFSA-US	PFSA/1.0 wt% CNTs-COO^−^	PFSA/1.0 wt% CNTs-SO_3_^−^
*ε*, mV	6	7	6
*D*, mV^2^	17	16	18
Drift, mV/h	4	insignificant	insignificant
Response time, min	<1		
pH (working range)	4.68–10.56		
*c*, M (working range)	1.0 × 10^−5^–1.0 × 10^−3^		
Stability, month	≥12		
LOD, M	1.8 × 10^−7^/5.8 × 10^−7^/1.8 × 10^−7^ (SAA^−^/SA/Na^+^)		
RSD, % (*n* = 4, *p* = 0.95)	5–18/4–13/4–20 (SAA^−^/SA/Na^+^)		
Relative error, %	5–10/6–17/1.7–9 (SAA^−^/SA/Na^+^)		

**Table 6:** The values of *t*-test and *F*-test criteria upon the recalibration of the multisensory system.

Membrane Composition	*b*_0_, mV		*b*_1_, mV/pSAA		*b*_2_, mV/pSA		*b*_3_, mV/pNa		*b*_4_, mV/pH		*t*-Test, *f* = 8, *p* = 0.95	*F*-Test, *f*_1_ = 3, *f*_2_ = 5, *p* = 0.95
	t	F	t	F	t	F	t	F				
PFSA-US	1.71	1.08	0.55	1.09	0.38	1.11	1.61	1.10	1.11	1.08	2.31	8.91
PFSA/1.0 wt% CNTs-COO^−^	0.33	1.06	0.53	1.04	0.09	1.10	0.39	1.07	0.44	1.03		
PFSA/1.0 wt% CNTs-SO_3_^−^	0.42	1.13	0.49	1.11	0.23	1.13	0.87	1.11	0.34	1.14		

**Table 7:** The analysis of the Sulfacyl sodium-SOLOpharm eye drops using the spectrophotometric technique.

Drug Pretreatment	*c*, M (Pharmaceutical Solution)		RSD, % (*n* = 4, *p* = 0.95)		*c*, mg/mL (Drug)	
	SAA	SA	SAA	SA	SAANa	SA
Dilution 1/25,000, acetate buffer (pH = 4.0)	(3.40 ± 0.02) × 10^−5^	-	0.3	-	200.6 ± 1.2	-
UV treatment, dilution 1/25,000, acetate buffer (pH = 4.0)	(3.11 ± 0.04) × 10^−5^	insignificant	1.5	-	184.5 ± 1.7	-

**Table 8:** The analysis of the Sulfacyl sodium-SOLOpharm eye drops using the multisensory system based on PFSA-US, PFSA/1.0 wt% CNTs-COO^−^, and PFSA/1.0 wt% CNTs-SO_3_^−^ membranes.

Drug Pretreatment	*c*, M (Pharmaceutical Solution)			RSD, % (*n* = 4, *p* = 0.95)			*c*, mg/mL (Drug)		Relative Error, %	
	SAA^−^	SA	Na^+^	SAA^−^	SA	Na^+^	SAANa	SA	SAANa	SA
UV treatment, dilution 1/1000	(8.1 ± 0.8) × 10^−4^	(6.7 ± 0.8) × 10^−5^	(8.9 ± 0.8) × 10^−4^	6	8	6	190 ± 18	11.5 ± 1.4	3	2

**Table 9:** Potentiometric sensors for the analysis of 4-aminobenzenesulfonic acid derivatives drugs.

Analyte *	Sensor Composition	Linear Range, M; LOD, M	Relative Error, %; RSD, %	Stability	Ref.
SAANa	PVC membrane with tetradodecylammonium SAA	1.0 × 10^−4.5^–1.0 × 10^−2^; 2.23 × 10^−5^	0.16–1.99; -	4 weeks	[69]
SAANa, SA	PFSA/PANI, PFSA/PEDOT	1.0 × 10^−4^–1.0 × 10^−2^; (4.1–7.2) × 10^−6^ (SAA^−^), 1.0 × 10^−5^ (SA)	1.2–1.4 (SAANa), 1.7–4 (SA); 6–7 (SAANa), 8–9 (SA)	≥12 months	[67]
	PFSA/CNTs–X	1.0 × 10^−5^–1.0 × 10^−3^; 1.8 × 10^−7^ (SAA^−^), 5.8 × 10^−7^ (SA)	3 (SAANa), 2 (SA); 6 (SAANa), 8 (SA)		This work
SDZ	PVC membrane with bis(triphenylphosphoranilidene)- ammonium SDZ	1.0 × 10^−5^–1.0 × 10^−2^; 4.36 × 10^−6^	0.7–2.6; 4.6–4.7	-	[70]
	PVC membrane with MIPs	9.0 × 10^−6^–1.0 × 10^−4^; 2.7 × 10^−6^	0.3–3.7; 0.6–1.4	2 months	[71]
SQX	Carbon paste electrode with 2,3,5-triphenyltetrazolium SQX	5.0 × 10^−6^–1.0 × 10^−2^; 3.0 × 10^−6^	-; -	-	[72]
SMX	PVC membrane with MIPs	1.0 × 10^−7^–1.0 × 10^−3^; 6.3 × 10^−8^	-; 0.25–0.36	3 months	[73]
SMX, TMP	PFSA/PANI with hydrothermal treatment	1.0 × 10^−5^–1.0 × 10^−3^; 1.4 × 10^−6^ (SMX^−^), 8.5 × 10^−8^ (TMP^+^)	4 (SMX), 5 (TMP); 5 (SMX), 6 (TMP)	≥12 months	[68]
	PFSA/CNTs–X	1.0 × 10^−5^–1.0 × 10^−3^; 3.5 × 10^−7^ (SMX^−^), 1.3 × 10^−7^ (TMP^+^)	4 (SMX), 5 (TMP); 6 (SMX), 7 (TMP)		[60]

* SDZ—sulfadiazine; SQX—sulfaquinoxaline; SMX—sulfamethoxazole; and TMP—trimethoprim.

## Data Availability

Not applicable.
